# Intermittent screening and treatment or intermittent preventive treatment compared to current policy of single screening and treatment for the prevention of malaria in pregnancy in Eastern Indonesia: acceptability among health providers and pregnant women

**DOI:** 10.1186/s12936-018-2490-3

**Published:** 2018-09-27

**Authors:** Jenna Hoyt, Chandra U R Landuwulang, Ansariadi, Rukhsana Ahmed, Faustina H Burdam, Irene Bonsapia, Jeanne R Poespoprodjo, Din Syafruddin, Feiko O ter Kuile, Jayne Webster, Jenny Hill

**Affiliations:** 1 Department of Clinical Sciences, Liverpool School of Tropical Medicine, Liverpool, UK; 2 Eijkman Institute for Molecular Biology, Jakarta, Indonesia; 3 Department of Epidemiology, School of Public Health, Hasanuddin University, Makassar, Indonesia; 4 Mimika District Health Authority, Timika, Papua Indonesia; 5 Timika Malaria Research Program, Papuan Health and Community Development Foundation, Timika, Papua Indonesia; 6 Department of Child Health, Faculty of Medicine, University Gadjah Mada, Yogyakarta, Indonesia; 7 Disease Control Department, London School of Tropical Medicine and Hygiene, London, UK

**Keywords:** Malaria in pregnancy, Intermittent screening and treatment, Intermittent preventive treatment, Acceptability, Pregnant women, Health providers, Malaria prevention, Anti-malarials, Dihydroartemisinin–-piperaquine

## Abstract

**Background:**

The control of malaria in pregnancy in much of Asia relies on screening asymptomatic women for malaria infection, followed by passive case detection and prevention with insecticide-treated nets. In 2012, Indonesia introduced screening for malaria by microscopy or rapid diagnostic tests (RDTs) at pregnant women’s first antenatal care (ANC) visit to detect and treat malaria infections regardless of the presence of symptoms. Acceptability among health providers and pregnant women of the current ‘single screen and treat’ (SSTp) strategy compared to two alternative strategies that were intermittent preventive treatment (IPTp) and intermittent screening and treatment (ISTp) was assessed in the context of a clinical trial in two malaria endemic provinces of Eastern Indonesia.

**Methods:**

Qualitative data were collected through in-depth interviews with 121 health providers working in provision of antenatal care, heads of health facilities and District Health Office staff. Trial staff were also interviewed. Focus group discussions were conducted with 16 groups of pregnant women (N = 106) to discuss their experiences of each intervention in the trial.

**Results:**

Health providers and pregnant women were receptive to screening for malaria at every ANC visit due to the increased opportunity to detect and treat asymptomatic infections. A primary concern for providers was the accuracy and availability of RDTs used for screening in the SSTp and ISTp arms, which they considered less accurate than microscopy. Providers had reservations about giving anti-malarials presumptively as IPTp, due to concerns of causing potential harm to mother and baby and as a possible driver of drug resistance. Pregnant women were accepting of all three interventions. Women in the IPTp arm were happy to take anti-malarials presumptively to protect themselves and their babies against malaria.

**Conclusions:**

The findings indicate that, within a trial context, malaria screening of pregnant women at every ANC visit ISTp was an acceptable strategy among both health providers and pregnant women owing to an existing culture of screening and treatment. The adoption of IPTp however would require a considerable shift in health provider attitudes and a clear communication strategy. By contrast, pregnant women welcomed the opportunity to prevent malaria infections during pregnancy.

**Electronic supplementary material:**

The online version of this article (10.1186/s12936-018-2490-3) contains supplementary material, which is available to authorized users.

## Background

Malaria in pregnancy has adverse consequences for both mother and baby. In Indonesia, 6.4 million pregnancies are exposed to malaria annually [1]. Malaria transmission varies from low to moderate with *Plasmodium falciparum* and *Plasmodium vivax* as the dominant species. Malaria infection in pregnancy in areas of low or unstable transmission, where women have little acquired immunity, is more likely to result in symptomatic malaria, severe disease and death of the mother or baby than in areas of moderate-to-high transmission. Both *P. falciparum* and *P. vivax* infections in pregnancy are associated with severe maternal anaemia, fetal loss and a reduction in mean birth weight or low birth weight babies [2, 3]. In Papua, asymptomatic and submicroscopic infections constitute two-thirds of detectable parasitaemia and are associated with a high risk of anaemia [4].

The control of malaria in pregnancy in most parts of Asia relies on screening asymptomatic women for malaria infection followed by passive case detection (PCD) and management of symptomatic malaria, and prevention with long-lasting insecticide-treated nets (LLINs) [5, 6]. Indonesia was the first country in Asia to introduce, in 2012, malaria screening as part of antenatal care (ANC) and to adopt dihydroartemisinin-piperaquine (DP) as first-line treatment for malaria in the second and third trimesters of pregnancy. The screening strategy consists of screening all pregnant women at the first ANC visit with either microscopy or rapid diagnostic tests (RDT), and treatment of test-positive cases with the nationally recommended anti-malarial (DP in 2nd or 3rd trimester and quinine in 1st trimester), henceforth referred to as ‘single screening and treatment (SSTp)’ [7]. However, a limitation of SSTp is the lack of prevention of re-infections later in pregnancy or missing asymptomatic cases in late pregnancy.

Weekly screening for malaria by microscopy and treatment of test positive women with effective anti-malarials substantially reduced maternal mortality from *P. falciparum* malaria in refugee camps on the Thai-Myanmar border [8], however such intensive screening programmes are unlikely to be feasible in routine health system settings. Recent trials in sub-Saharan Africa [9–12] found intermittent screening and treatment in pregnancy (ISTp) with RDTs at every scheduled ANC visit (average of 3–6 visits) and treatment of positive cases with artemisinin-based combination therapy (ACT) was acceptable and potentially feasible [13–16]. However, recent results from the clinical trials indicate that, with the current generation of malaria RDTs, ISTp was not a suitable alternative strategy to intermittent preventive treatment with sulfadoxine–pyrimethamine (IPTp-SP), even in areas with high prevalence of SP resistance in East and Southern Africa [12].

A cluster-randomized controlled trial was undertaken between May 2013 and November 2016 to compare the efficacy of IPTp or ISTp with DP versus the current policy of SSTp with DP in the 2nd and 3rd trimester in preventing the adverse outcomes of malaria in pregnancy in an area of relatively low *P. falciparum* and *P. vivax* transmission in eastern Indonesia. The objective of this study was to assess the acceptability and perceptions of health providers and pregnant women towards ISTp and IPTp compared to SSTp in the context of the clinical trial. Should the efficacy trial indicate that a shift to either ISTp or IPTp is superior to the current strategy of SSTp in preventing the adverse outcomes of malaria in pregnancy, then understanding the acceptability among pregnant women and health providers of the different strategies will help inform programme implementation and the communication strategy needed to promote effective delivery by providers and facilitate uptake by pregnant women.

## Methods

A qualitative study was conducted in two rural districts in Eastern Indonesia, in South West Sumba District and in Timika sub-district of Mimika District, the same study sites where the main trial was conducted. In depth interviews (IDIs) with health providers in South West Sumba were conducted between May and July 2015 and in Mimika between February and April 2016. Focus group discussions (FGDs) with women at both sites took place in December 2016.

### Study sites

Mimika District lies in the south of Papua Province, Eastern Indonesia, covering 19,952 square kilometres consisting of highlands and lowlands. It has a rapidly growing population of an estimated 182,000 (2010 census) Papuan and non-Papuan Indonesians, with the main occupations in commerce, business, and retail. The prevalence of parasitaemia among pregnant women at delivery is 16.8% (58% *P. falciparum* infections, 34% *P. vivax*, and 8% mixed infections), with 35% of these infections being associated with fever [3].

South West Sumba District is situated in a low volcanic island (Sumba) occupying 1445 sq km in East Nusa Tenggara Province. It has a population of 283,818 (2010 census) of mainly native Sumbanese Indonesians and the main occupation is subsistence farming. Malaria on the island is seasonal, with a dry season from May to November and a wet season from December to April. In 2012, the antenatal prevalence of infection in pregnancy was 3–5% by RDT or microscopy and 7% by Polymerase chain reaction (PCR) [17].

The main health facilities which deliver ANC services to pregnant women are hospitals, community health centres (known in Indonesian as *puskesmas*) covering about 30,000 people, sub health centres (*pustu*) which serve about 2–3 villages and 2000–3000 population, and health posts (*posyandus*) or community integrated services including antenatal care, which are held monthly or bi-monthly in villages. The trial selected ANC facilities which had more than 10 new pregnancies per year and were located within 1.5 h drive from the study offices in each site [Ahmed, personal communication]. ANC attendance in Mimika and South West Sumba districts is similar, with 84% and 87% of pregnant women making at least one ANC visit, respectively [18, 19]. Only a third of women (32%) make at least 4 ANC visits in Mimika; data for West Sumba was not available. The key indicators for ANC utilization captured in the ANC registers are: ‘K1 pure’ which measures 1st ANC visit in the first trimester, and ‘K1 contact’ which measures first ANC visit in 2nd or 3rd trimester.

### Trial context

The main trial was an open-label three-arm parallel-group matched cluster-randomized controlled superiority trial in two sites (Mimika District and South West Sumba) comparing the efficacy, safety and cost-effectiveness of IPTp and ISTp *vs* the current SSTp strategy. DP was used in all 3 arms (Trial registration no. ISRCTN34010937). The study selected health centres (*puskesmas)* and their related health posts (*pustu* or *posyandu*) in each trial site to enroll pregnant women. Recruitment began in May 2013 and the last case follow-up of infants was completed in November 2016. Participants received one of the three interventions at each scheduled ANC visit during the 2nd and 3rd trimesters of pregnancy according to the randomly allocated cluster. Visits were scheduled every 4 weeks (± 3 days) with the total number of ANC visits ranging from 3 to 6 (median 3). The trial used First Response Malaria Ag pLDH/HRP2 Combo RDTs (Premier Medical Corporation Pvt Ltd, India) as the point of care test for malaria screening.

### In-depth interviews with health providers

All health facilities involved in the clinical trial were included in the study, comprising 10 health centres (*puskemas*) and their related health posts (*posyandus)* in South West Sumba and 7 health centres in Mimika plus staff from the District health Office (DHO) and staff involved in the clinical trial from each district.

IDIs were conducted with health providers who were purposively selected according to their role in the delivery of antenatal services, including midwives, doctors, laboratory staff, pharmacists, head of drug store, and the facility head. In Sumba, facility and drug store heads had no involvement with antenatal service delivery and were not selected. The consent of the head of the health facility was first sought and, subject to consent, staff selected for interview were then invited to give written consent to be interviewed at a convenient time.

Interviews were conducted using an interview guide to explore the following themes: (1) their acceptability of SSTp, ISTp and IPTp; (2) their perceptions of pregnant women’s acceptability of each trial arm; (3) their perceptions of the feasibility of implementing each of the interventions at scale, and (4) their recommendations on factors to be considered to ensure effective implementation. Interviewers adopted an iterative process whereby any emergent themes raised by participants relevant to the objectives of the study were explored further and/or included in subsequent interviews. The interviews were conducted in Indonesian in both sites by trained staff, and digitally recorded.

### Focus group discussions with pregnant women

FGDs were conducted with participants in both South West Sumba and Mimika districts in December 2016, after follow up of trial participants was completed so that experiences from a full pregnancy history could be obtained for each woman. Women were purposively selected using the study database to represent views and experiences from each of the three trial arms and to ensure a mix of women in the two screening arms who tested positive and negative to malaria during the trial. A total of 16 FGDs were carried out, 7 in South West Sumba and 9 in Mimika comprised of women from 6 different groups: (1) ISTp arm, RDT negative, (2) ISTp arm, RDT positive, (3) SSTp arm, RDT negative, (4) SSTp arm, RDT positive, (5) IPTp arm, and (6) a heterogenous group with women from all three interventions groups. Due to low malaria incidence in both sites, FGDs with RDT positive women were not conducted in Sumba, and only one FGD with RDT positive women for both ISTp and SSTp arms was conducted in Mimika.

### Data management and analysis

In-depth interviews and FGDs were transcribed and translated into English by external translators and checked by the trial Research Assistant for any diversion in meanings of the content. The transcripts were then entered into NVivo Version 11.2 for data management and analysis. The data were coded by J Hoyt and analysed by J. Hoyt, J. Hill and J. Webster; any conflicting views were discussed among the team until a consensus was reached. A similar framework to that used in a previous study [14] was used to structure the interview theme guide was also used for coding and initial analysis of the IDIs. Each of the intervention arms were further coded according to the WHO health systems building blocks [20]: financing, governance, health information, human resources, products and technology, and service delivery. The framework used to analyse the FGDs comprised of: experiences and acceptability of the interventions as a whole and their individual components (i.e. malaria testing and anti-malarials); and adherence to anti-malarials for treatment (SSTp and ISTp) and prevention (IPTp). A content analysis approach was used to derive emergent themes and sub-themes considered important to the acceptability of the trial interventions, and relevant quotes used to enrich and support the findings.

## Results

Interviews were conducted with 121 health providers; 50 health providers from 10 health centres, four DHO staff and two trial staff in South West Sumba (n = 56), and 56 health providers from 7 health centres, five DHO staff and four trial staff in Mimika (n = 65). A list of cadres interviewed can be found in Table 1. A total of 106 women participated in 16 FGDs in the two sites. Summary characteristics of the women can be found in Table 2. The women were aged between 16 and 44 years and most were married (88%) and had reported between 1 and 8 pregnancies. Key themes emerging from the analysis of data from health providers and pregnant women are presented here together with illustrative quotes, and a summary of themes from both groups of participants is presented in Table 3. Quotes supporting the sub-themes are provided in Additional files 1 and 2.

**Table 1:** Health provider cadres interviewed at Sumba and Mimika sites

	Sumba	Mimika	Total
Head of puskesmas^a^	0	7	7
Doctor	10	7	17
Lab technician	10	7	17
Malaria coordinator	11	8	19
Midwife coordinator	11	8	19
Village midwife	10	7	17
Pharmacist	1	8	9
Head of drug store	0	8	8
Head of DHO	1	1	2
Trial staff	2	4	6
Total	56	65	121

^a^Puskesmas—health centres

**Table 2:** Summary characteristics of pregnant women participating in the focus group discussions

Site	Trial arm^a^	Number of focus group discussions (FGDs)	Number of participants (n)	Average age (range)	Marital status (n)	Average number of pregnancies (range)
Sumba	IST negative	2	16	30 (24–42)^b^	Married (10), single (6)	3 (1–8)
Mimika	IST negative	2	16	29 (20–40)^c^	Married (14), single (2)	3 (1–5)
Sumba	SST negative	2	11	34 (24–42)	Married (8), single (3)	4 (1–6)
Mimika	SST negative	2	12	27 (18–39)	Married (12), single (0)	2 (1–8)
Mimika	IST positive	1	7	25 (16–32)	Married (7), single (0)	2 (1–5)
Mimika	SST positive	1	6	33 (25–42)	Married (6), single (0)	4 (2–6)
Sumba	IPTp	2	10	27 (20–32)	Married (9), single (1)	2 (1–4)
Mimika	IPTp	2	16	26 (16–40)	Married (15), single (1)	2 (1–6)
Sumba	Heterogenous	1	6	33 (25–44)	Married (6), single (0)	3 (2–4)
Mimika	Heterogenous	1	6	24 (19–37)	Married (6), single (0)	2 (1–4)

^a^Due to low malaria incidence FGDs with IST or SST positive women did not take place in Sumba

^b^12 out of the 16 women reported their age

^c^15 out of the 16 women reported their age

**Table 3:** Themes on the acceptability of ISTp and IPTp among pregnant women and health providers

Theme	Pregnant women	Health provider
SSTp		
Major themes	Happy to be screened for malaria	Early detection and treatment is very important Stocks of RDTs were not stable Malaria in pregnancy can be asymptomatic
Minor themes	Would prefer ISTp over SSTp	Screening once is not enough Testing when symptomatic is sufficient
ISTp		
Major themes	Happy to be tested & know malaria status Happy to be tested monthly Happy to be tested even when asymptomatic	Screening at every visit is a good strategy Women can contract malaria at any time in the pregnancy Women are happy to be screened for malaria^a^
Minor themes	Prefer testing only when symptomatic	Some women may not like monthly testing^a^ Asymptomatic screening at first visit is sufficient
RDTs		
Major themes	Happy to receive results right away Don’t mind the finger prick	Results are not always accurate RDTs don’t detect all malaria species Supply is not stable, frequent stock-outs Fast and easy to use Good for use at village posts, where lab services/electricity not available
Minor themes	Afraid of needle/blood loss	Prefer RDT over microscope
IPTp		
Major themes	Happy to take drugs to prevent malaria and be healthy (even with side effects) Will take the drugs when reassured there is no harm to baby, given by trusted provider	Women should be tested before they are given anti-malarials Taking medication during pregnancy when there is no disease could cause harm Could increase drug resistance
Minor themes	Prefer testing before taking drugs Hesitant at first to take drugs but did so for baby’s health	Prevention is a good idea, if the drug is safe Some women may not want to take drugs if they are not sick^a^ Some women in the IPTp arm refused 2nd dose of IPTp
DP anti-malarial		
Major themes	Experienced side effects of nausea, dizziness, sleepiness Completed my treatment/IPTp dose	Effective treatment for malaria Well tolerated by women Some reported side effects of nausea, vomiting, dizziness
Minor themes	Experienced vomiting Did not complete my treatment/IPTp dose	Some refused to take subsequent IPTp doses DP is hard and bitter
Service delivery		
Major themes		Screening should be carried out at health posts, more accessible to women Midwives should use RDTs at health posts to screen for MiP Midwives should be able to give anti-malarials to women when necessary
Minor themes		Drugs should only be prescribed by doctors or nurses under supervision

*DP* dihydroartemisinin-piperaquine, *IPTp* intermittent preventive treatment, *ISTp* intermittent screening and treatment, *MiP* malaria in pregnancy, *RDTs* rapid diagnostic tests, *SSTp* single screening and treatment

^a^Indicates when it is a health provider perception of how pregnant women feel

## Health providers

### Acceptability of current policy—SSTp

Health providers in South West Sumba and Mimika were supportive of the current policy of SSTp. Many cited the importance of early detection and treatment of malaria in pregnancy as a key benefit of the strategy. There was acknowledgment that women are often asymptomatic during pregnancy thus screening at the first ANC visit regardless of symptoms was important to protect both mother and baby.

Health providers in Sumba discussed the challenges posed by the lack of resources needed to carry out the SSTp strategy in the national programme and reported that, without reliable supplies, they were unable to implement the policy consistently. Frequent reports of RDT stock-outs meant SSTp was often not being implemented at the health posts, which have no access to microscopy. These reports of stock-outs refer to time periods outside the trial, since RDTs and DP were supplied to all participating facilities during the trial.

One village midwife from Mimika mentioned that screening once in pregnancy was not enough to offer full protection to mother and baby, while two participants felt that testing only symptomatic women would be sufficient.

### Acceptability of trial interventions—ISTp

There was strong support for ISTp from the health providers across all sites. The main benefit reported was that screening at every ANC visit would provide women with more complete protection as they could contract malaria at any time during their pregnancy.

“R: *As I know, sometimes the pregnant woman who had malaria screening and treatment at K1 [1st ANC visit] believed that she had no malaria anymore. However, we were not sure whether she got malaria infection when she went home [after she was identified negative during the first visit at clinic]. That’s why it will be better if the pregnant woman is screened at every antenatal care visit every month so we can make sure that the pregnant woman is free from malaria until the day of delivery*.” Malaria coordinator, SUMBA

For some, ISTp was the best option among the three interventions for reasons including a preference for testing before giving anti-malarials, and continuous testing throughout pregnancy provided more comprehensive coverage.

However, not all participants felt that testing at every visit was the best option. Some providers worried about a reliance on RDTs, which were often not available through the national programme, and about insufficient resources generally, including reagents for microscopy. Other providers felt that women might complain about being tested at every visit and one midwife commented that they might be too busy to screen women at every visit. Several providers said the current strategy of screening at the first ANC visit and subsequently if symptomatic (SSTp) was sufficient.

“I: *Is there any problem so far in screening test using RDT method? R: The unavailability of RDT kit itself is a problem. I didn’t know what causes it*.” Village midwife, SUMBA

### Acceptability of trial interventions—IPTp

Health providers at both sites expressed concern with regards to the IPTp strategy. Most frequently reported across all cadres was the assertion that women should be tested for malaria before being given anti-malarials and, closely related, that taking medication in pregnancy, particularly if there was no disease present, could cause harm to mother and/or baby. Malaria coordinators, doctors and one head of facility worried that the provision of preventive anti-malarials could accelerate resistance to key drugs.

“I: *What do you think about giving anti*-*malarial drugs without screening? R: It is not allowed. The diagnosis of malaria has to be confirmed first before we give the drug. We can’t make diagnosis of malaria by bare eyes. We cannot determine malaria cases by looking at their symptoms because the symptoms may be due to another disease*.” Malaria coordinator, MIMIKA

Several midwives, a doctor and one facility head were open to the idea of giving preventive doses of anti-malarials to women under the belief that prevention was a good idea. Furthermore, providers who were asked specifically about giving drugs without screening as “preventive” within the context of a clinical trial were more likely to show acceptance towards IPTp than those who were asked the question out of context.

“I: *Relating to the IPTp method that we should give anti*-*malarial drug without screening, what do you think about that? R: I am not sure whether it is safe or not if we [just] administer [malaria] drug without assessing whether they have malaria symptoms I: So, do you disagree with that method? R: However, as long as there is a guideline for this method, I have no problem to implement [IPTp]*” Village midwife, SUMBA

Among providers who did not disagree with IPTp many were still hesitant about the strategy with concerns about women not wanting to take medication if they don’t feel ill, safety of the drugs, additional communication with women and increased workload to follow-up adherence to drugs. A few midwives recalled the earlier practice of chloroquine prophylaxis during pregnancy and their responses were mixed as to whether they felt it was a good programme or not.

### Perceptions of pregnant women’s attitudes towards trial interventions

Although providers were not directly asked about pregnant women’s acceptability towards elements of the trial interventions, some participants volunteered their perceptions on how women felt about malaria screening and the three trial arms. In general, they felt women were happy to be screened for malaria, despite a few women who were concerned with the amount of blood being taken (referring to the venous blood taken at the first ANC visit). One trial staff was asked if they would be charged for the test and one midwife reported hearing that some women in the ISTp arm complained about being tested every visit.

Trial staff involved in the IPTp arm reported that some women experienced side effects after taking DP including dizziness, nausea and vomiting. Staff said they had explained to women that the effects would not last long and highlighted the importance of protecting against malaria. Two trial staff at Mimika facilities reported that some women in some of the clinics refused to take subsequent doses of IPTp-DP when they returned to the ANC due to side effects.

Two midwives and one trial staff expressed concern that some women might not be willing to take medications if they were not ill. Other reactions by women in the IPTp-DP arm, as reported by health providers, included asking if DP was safe to take during pregnancy and comments that DP was hard and bitter. Two trial staff reported that informing women of the protective effects of IPTp-DP had motivated some women to take the drugs.

### Acceptability of elements of trial interventions—RDTs and DP

At both Sumba and Mimika facilities and across health provider cadres, participants expressed serious doubts with regards to the accuracy of RDTs, with many reporting that they did not trust negative RDT results and would request a re-test using microscopy if women were symptomatic. Another limitation was that RDTs were perceived to only detect falciparum or mixed infections, leaving ovale or vivax infections undiagnosed. RDTs were considered useful as an alternative when microscopy was not available or during power outages. However, participants were quick to add that they preferred microscopes, as they were more accurate.

A smaller number of providers reported that RDTs worked well and many acknowledged that they were easy to use and provided faster results. Despite these benefits there were still concerns regarding accuracy. One doctor at a Sumba site stated a preference for RDTs saying that microscopy was subject to human error.

Availability of RDTs was a major issue for the current SSTp policy, with many participants of the FGDs and IDIs reporting frequent stock outs at their facility. These logistical issues were particularly pronounced at facilities in Sumba, where supply issues in the national programme negatively affected overall acceptability of RDTs as a screening tool and to ISTp as a potential alternative strategy.

Health providers reported that some women complained about nausea, vomiting and dizziness after taking DP. Two midwives reported cases where a woman became “limp” after taking DP and there was one report of fatigue and headache. However, many providers said they received no reports of any negative side effects due to DP, and a few participants (mainly doctors and pharmacists) said they preferred DP as it had fewer side effects and a shorter dosing schedule than quinine. One malaria coordinator and district level officer said that some midwives were nervous to give DP, fearing bad side effects.

“I: *Any problem in DHP prescription? R: So far, there is none, and women often reported that they tolerated DHP and the experience of nausea was minimal. Patients feel comfortable to take DHP and recovered quickly after taking DHP*.” Doctor, MIMIKA

Despite being first-line treatment for malaria in pregnancy and for non-pregnant patients, frequent stock outs of DP were widely reported by providers at all facilities in Sumba and at a few facilities in Mimika. The reason behind the stock outs was not well understood. Some facilities seemed to have a steady stock of DP, and a few participants reported that they had received DP either close to or past the expiry date.

### Views on service delivery options of trial interventions

There was general approval among midwives for malaria in pregnancy screening to be carried out at rural health posts ‘*pustu*’ or ‘*posyandu*’, instead of at the health centres (*puskesmas*) as the health posts were more accessible to women, reducing transportation costs over long distances. There was strong acceptance among providers for midwives to screen pregnant women at the health posts with RDTs. However, microscopy was preferred for screening women at the health centres due to availability and accuracy. Midwives were generally more accepting of RDTs stating that the results were fast and the device easy to use.

Whilst it is not standard practice for midwives to give prescription drugs, there was cross-cadre acceptance for trained midwives to be allowed to give anti-malarials when necessary given the lack of pharmacists. A few providers in both sites felt drugs should only be prescribed by doctors, or nurses under supervision.

## Pregnant women

### Acceptability of screen and treat strategies (SSTp and ISTp)

Pregnant women were overwhelmingly positive about the benefits of being tested for malaria, knowing their status and receiving appropriate treatment. Furthermore, there was broad acceptance for regular testing despite being asymptomatic. Among women in the ISTp arm, most were happy to have regular blood tests and some women in the SSTp arms indicated a preference for monthly testing, even without symptoms, over SSTp. Encouragingly, several women said they would accept regular malaria tests in a future pregnancy, outside of a trial setting. A few women reported being initially afraid about the blood test but understood its importance for their health and that of their baby. A few women in both Sumba and Mimika reported a preference for testing only when symptomatic.

“I: *What did you feel when your blood was drawn? P8: I was shocked. I: Do you like blood drawing from your finger or not? P8: Yes, I do because then I could know whether I have malaria or not. Should we have malaria, then the drugs will be given. If malaria is not detected in our system then we are just happy because we check for it monthly*.” ISTp negative, MIMIKA

Women from the RDT positive groups reported completing the treatment dose of DP except one woman who reported stopping after 1 day because she felt better and was afraid to keep taking the drugs.

### Acceptability of anti-malarials for prevention (IPTp)

The majority of women were happy to take preventative doses of DP as part of the IPTp strategy, despite experiencing side effects. A few women said they hesitated because of fear of side effects but took them “for the health of the baby”. Commonly reported side effects after taking DP included nausea, dizziness and drowsiness, with a few women saying they vomited shortly after taking the drug. Some women discussed a preference for eating food before taking the dose at home as a way of preventing nausea. A few women reported a preference for testing before taking anti-malarials, even when described as “preventative”, and a small number of women said they felt confused about taking DP because they didn’t have malaria. However, the majority of women supported the preventative strategy if it was explained clearly, brought no harm to the baby and was administered by a trusted health provider. Most women reported completing the remaining two IPTp doses at home with no problems. A few women said they did not complete the dose due to size of DP, not having malaria or, in one case the woman’s husband used them to treat himself.

“I: *So, you took the medicine that you knew was for malaria prevention? P2: Yes, because we knew it’s for malaria prevention, and more for our baby. Moreover, if my mum is also there, yes I had to take the drugs. I: So for the next pregnancy, will you take the drugs to for malaria prevention? All: Yes, I will. I: Why would you take the drug [malaria drug]? P5: Yeah, we want to take it, to keep our babies and ourselves healthy and free from malaria. I: What about if you still experience dizziness? P5: That’s alright. We need to ensure that mother and baby are healthy*.” IPTp, MIMIKA

## Discussion

This represents the first study evaluating provider and user acceptability of ISTp and IPTp with DP in the Asia region, and in a country where the current policy is SSTp. The qualitative data presented here demonstrates that among providers a shift from SSTp to ISTp would be a simpler conceptual step but that, with the proper communication strategy, the IPTp strategy could be supported. Among pregnant women, both ISTp and IPTp were widely acceptable alternatives to SSTp. This study adds rich data on provider and user attitudes towards malaria in pregnancy control strategies and offers key insights for policy-makers regarding potential opportunities and challenges were Indonesia to shift from a screen and treat-focused policy to one that relies on presumptive use of anti-malarials.

Health providers were very receptive to ISTp as it provides more frequent opportunities to detect asymptomatic infections over the course of pregnancy than SSTp. Many providers felt this was more comprehensive and better for mother and baby than the current SSTp policy. The primary concern with the ISTp strategy during the trial was the reliance on RDTs for screening. RDTs were considered less accurate than microscopy and their unavailability due to regular stock-outs in the national programme was a limitation, particularly in Sumba. This concern is not unfounded as the current SSTp strategy relies on a stable supply of RDTs procured with external donor funding and is subject to fluctuations in funding levels. There is mounting evidence from studies elsewhere that health providers regard RDTs as less accurate than microscopy but acknowledge their usefulness where resources are limited. Health providers in western Kenya described microscopy as the gold standard, while RDTs were considered useful in settings without laboratories or electricity but had limited sensitivity and specificity [14]. Lack of trust in negative RDT results prompted some providers to advise re-checking RDT results using microscopy, as found in an evaluation of the SSTp strategy in West Sumba and Mimika [21]. Provider mistrust was also reported in Uganda, where only 49% of health workers believed a negative result to be true [22]. Concerns regarding RDT accuracy may be warranted as the results from the clinical trial indicate that, at current levels of RDT sensitivity, subpatent cases were missed when RDTs were compared with more sensitive molecular testing (Loop mediated isothermal amplification and PCR) such that ISTp failed to detect more cases of malaria in pregnancy than SSTp despite more frequent testing [Ahmed, pers. comm.].

Pregnant women were happy to be screened for malaria at regular intervals even when asymptomatic. More importantly women perceived screening and treatment as part of a “complete” ANC package and necessary for maintaining good health during pregnancy. Women frequently listed health as a primary motivating factor for attending ANC and accepting the services on offer, with some women indicating that trust in the health provider reassured them when offered an intervention. A similar study in Ghana found that women were less concerned by which intervention was offered, accepting both ISTp and IPTp, since they were part of ANC services that they trusted and viewed positively [16, 23]. In this study, acceptability towards regular screening for malaria was high amongst both users and providers, likely due to the existing culture of screening created by the current SSTp policy in Indonesia. In contrast, a study from Northern Ghana assessing acceptability to ISTp compared to current policy of IPTp found that pregnant women disliked frequent blood testing. Some women reportedly asked for IPTp-SP to be given instead of undergoing regular screening [24]. However, a study in western Kenya, where IPTp is current policy, found that women accepted ISTp and understood that they would only receive anti-malarials if they tested positive [14]. This reflects women’s trust in the interventions provided at ANC and supports the concept that women accept what is on offer.

The unavailability of RDTs in the context of the current SSTp policy was undoubtedly a concern among providers but it also raised questions about the feasibility of implementing ISTp, a strategy that requires more frequent testing and increased numbers of RDTs. This challenge affected the overall acceptability of ISTp as an alternative strategy. With RDTs being a core element of rolling out ISTp to village health posts, supply issues would need to be addressed for health providers to fully back a policy change. Furthermore, even with a regular supply chain the issue of RDT accuracy would still present a significant barrier to acceptability. The effect of implementation issues on provider acceptability and use of new technology has been described by a study in Uganda exploring provider experiences with RDTs in resource-limited settings [22]. Health providers in western Kenya expressed reservations with regards to the reliance of the ISTp strategy on RDTs, noting that they were expensive and the results often unreliable [14].

As frontline ANC staff providing services in the villages, midwives were more concerned with providing essential services that are accessible to women than the technical efficacy of RDTs. This was demonstrated by midwives’ preference for screening to be carried out at the rural health posts, as they were more accessible to pregnant women than the larger health centres. Health providers in general struggled with the concept of giving pregnant women drugs without a positive diagnostic test. Primarily, giving anti-malarials presumptively was against current national policy, but there were also concerns about causing potential harm to mother and baby and of driving drug resistance. This view is a sharp contrast to studies from Africa where IPTp is widely used to prevent malaria in pregnancy, and health provider acceptance of prevention over treatment is strong [13, 15]. In contrast, in Sumba and Mimika the concept of giving drugs before receiving a positive test was seen as a form of malpractice with some providers emphatically stating they would never do such a thing. The current policy of SSTp has invariably fostered a culture of *screening before treatment* and as such a strategy shift from SSTp to ISTp was conceptually easier to accept than a shift to IPTp. User and provider acceptance towards ISTp will likely be influenced by whether the existing policy is based on screen-and-treat and/or presumptive treatment; should the new policy require a major shift in approach, such as from SSTp to IPTp, there could be resistance among providers.

A key point needing further investigation is whether health providers perceived the use of anti-malarials for *prevention* in the same negative light that they seemed to view the general dispensing of medication without confirmation of a test result. Some insight into this question may be seen in health provider’s reactions to differences in the way the interviewer structured the question about IPTp. Providers were more open to the concept of IPTp if the interviewer described giving anti-malarials for “prevention” within the context of a clinical trial instead of simply asking if they would ever give anti-malarial drugs without screening first. This could represent bias within the trial context, but perhaps it demonstrates that with the appropriate communication, training and policy guidelines health providers may potentially be receptive to IPTp as a preventative strategy, but this would require careful monitoring. It further highlights the need to develop an effective behaviour change communication strategy to foster acceptance to IPTp among health providers prior to any new policy implementation. Interestingly, health providers in areas with the highest prevalence of parasitaemia in pregnancy in Papua appeared more receptive to IPTp as a control strategy, perhaps based a on perceived greater need, an area worth further exploration. This perception was supported in the trial results, which showed that IPTp was more effective than SSTp in reducing the incidence of malaria during pregnancy and at delivery in the higher transmission sites in Papua [Ahmed, pers. comm.]. Crucially, for IPTp to be feasible midwives will need to dispense anti-malarials at health posts, a concept that was acceptable to many providers, particularly in Mimika district.

Interestingly, the women in this study did not share this apprehension for taking anti-malarials presumptively, even when they experienced side effects, with many favouring prevention over cure. This could be due to concerns about the effects of malaria in pregnancy and previous experiences of malaria infections. Health providers frequently reported that women would object to taking anti-malarials without testing positive and, while some women in the IPTp arm expressed hesitation over concerns of side effects or harm to the baby, the vast majority were happy to take the preventative dose and said they would do so again in a future pregnancy.

Acceptance towards DP as treatment for RDT positive cases within the ISTp trial arm was strong among health providers, who felt it was well tolerated by women and effective in treating malaria. Pregnant women in the IPTp arm reported side effects including nausea, vomiting and dizziness but this did not seem to act as a barrier to use. These side effects have been reported elsewhere in Africa in trials of DP as an alternative drug for treatment and for IPTp [12, 14]. However, even mild side effects have contributed to the lack of effective coverage for IPTp-SP across Africa despite decades of implementation with several studies reporting side effects as a barrier to uptake of IPTp [25, 26]. Importantly, DP was seen as an effective treatment and preferable due to the shorter dosing regimen when compared with quinine. Regardless of the malaria in pregnancy strategy, supply chain issues for DP will need to be addressed as stock outs for first line treatment in the general population were widely reported in the national programme in Sumba.

### Limitations

The opinions and experiences of health providers and pregnant women interviewed within the context of a trial cannot be generalized to non-trial settings. Social desirability bias may have influenced provider responses regarding aspects of the trial and their attitude towards administering anti-malarials presumptively in the IPTp trial arm. Similarly, there could be a bias towards reporting positive opinions about the interventions used in the trial among pregnant women. Women selected to participate in the FGDs could differ from those who did not participate in a way that excludes some views from the data, however, the researchers agreed that saturation of women’s opinions was reached during the analysis of the FGDs. The results from this study have limited generalizability beyond the population sampled but steps were taken to explore a wide variety of attitudes and opinions by interviewing participants across different health provider cadres and pregnant women across all intervention arms.

## Conclusions

Acceptability of screening women at every ANC visit (ISTp) was high among providers and pregnant women, owing to an existing policy and culture of screening women at ANC and providing treatment based on a positive diagnosis. However, more sensitive RDTs and a reliable supply chain will be required to garner full approval among health providers. Providers were hesitant to support giving anti-malarials presumptively (IPTp) suggesting a strong campaign of effective communication and training would be required to achieve a conceptual shift. Encouragingly, women were happy with any opportunity to prevent malaria in pregnancy and accepted IPTp as part of a package of valued ANC services.

In the context of this study, replacing SSTp with IPTp would appear to require a more challenging conceptual shift for providers but its superior efficacy and non-reliance on RDTs means that providers may be persuaded to see it as a more realistic strategy in higher transmission settings in Indonesia.

## Additional files

**Additional file 1.** Quotes from IDIs with health providers on major and minor themes”. Quotes from health providers to support the analysis of the themes and sub-themes presented in the results.

**Additional file 2.** Quotes from FGDs pregnant women on major and minor themes”. Quotes from pregnant women to support the analysis of the themes and sub-themes presented in the results.
