# Supplementary material for: Intermittent screening and treatment or intermittent preventive treatment compared to current policy of single screening and treatment for the prevention of malaria in pregnancy in Eastern Indonesia: acceptability among health providers and pregnant women
Source: Malar J. 2018 Sep 27;17:341. doi: 10.1186/s12936-018-2490-3 (PMC6161378; doi:10.1186/s12936-018-2490-3)
Supplement: Supplementary file 1 — Additional file 1. Quotes from IDIs with health providers on major and minor themes”. Quotes from health providers to support the analysis of the themes and sub-themes presented in the results. [file 12936_2018_2490_MOESM1_ESM.doc]

Additional File 1: Quotes from health providers on major and minor themes

| **Theme** | **Sub themes** | **Quotes** |
| --- | --- | --- |
| **SSTp** |  |  |
| **Major themes** | Early detection & treatment is very important | “I: Do you think that the screening program is quite effective program or do you have anything that you want change in that program? R: Screening program for pregnant woman or? I: For pregnant woman. R: I think that program is good because we can take action to anticipate malaria infection in pregnancy as soon as possible.” Doctor, SUMBA  “I: what do you think about every pregnant woman who comes to health facility should be tested for Malaria? R: I agree with that. That is same with my suggestion. I: So every pregnant woman in the first visit should be tested for malaria? R: Pregnant woman with or without symptom should be tested so we can detect the malaria and give the treatment earlier.” Malaria coordinator, MIMIKA  “I: What do you think about screening test every ANC visit? R: That’s great idea. So we can detect earlier the symptom of malaria.” Midwife coordinator, SUMBA |
|  | Stocks of RDTs were not stable | “I: Do you think it needs a change? R: Don’t need to change, just need some improvement in RDT procurement since there are no more stock available at the village. If only we had RDT then we could’ve performed an RDT.” Village midwife, SUMBA  “I: Ee... You agree with that. Is there anything you want to change? R: Ee... If it’s possible I want to improve it… I: What do you want to improve? R: I think it’s good so it will be better if we can improve it, for example we haven’t done this program at Posyandu [health post] because sometime we don’t have enough RDT to do that at Posyandu.” Midwife coordinator, MIMIKA |
|  | Malaria in pregnancy can be asymptomatic | “R: To be honest, I would love to, I thought when it came to pregnant women, she has to be tested whether she has symptoms or not. I: what makes you think so? R: malaria is, even if there are no symptoms shown but the patient may have the parasites inside the body I: ah, yes. R: so that’s it.” Lab tech, SUMBA  “I: Do you think that the malaria screening program is the best method for malaria prevention in pregnancy? R: Yes, I think Malaria screening is the best method for malaria prevention in pregnancy I: Why? R: It is because in Timika people already have immunity for malaria so although the patient has no symptom but actually the patient has parasite inside her/his body….” Village midwife, MIMIKA |
| **Minor themes** | Screening once is not enough | “I: in your experience, or in your observation so far for single screening and treatment. As you said about the pregnant woman that visits for the first time would be screened, examined, but not in the following visit – only if symptoms presents, and do you think that this is the best approach in preventing malaria in pregnancy? R: to say that it is the best, I don’t think it suits, not yet. Because the test is only performed once when they visited. But at least there is an effort. It’s just one of the way, probably there is more – there are more ways, like the one that the team did for the trial, it’s pretty good. To perform the test once, I think it’s not yet maximum. I: why is it not yet maximum? R: because it is only one time, while pregnancy is in long period of time, 9 months. So it could be after we examined her, she would be bitten by mosquito when she got home.” Village midwife, MIMIKA |
|  | Testing when symptomatic is sufficient | “I: Do you think that it will be good if every pregnant woman who comes for ANC visit should be screened for Malaria? R: I think not all pregnant women should be screened for malaria unless they have malaria symptom. If the pregnant woman who comes here has malaria symptoms, we have to do malaria screening to her immediately so she could be treated properly so that Malaria couldn’t give bad impact for her pregnancy.” Pharmacist, MIMIKA |
| **ISTp** |  |  |
| **Major themes** | Screening at every visit is a good strategy | “R: As I know, sometime the pregnant woman who got malaria screening and treatment at K1 [1^st^ ANC visit] believed that there was no malaria in her body anymore. But we were not sure whether she got another event of malaria infection at home or not. That’s why it will be better if the pregnant woman is screened at every antenatal care visit every month so we can make it sure that the pregnant woman is free from malaria until the day of delivery.” Malaria coordinator, SUMBA |
|  | Women can contract malaria at any time in the pregnancy | “I: what do you think about the screening test perform in every ANC visit R: I’m fine with it, even in this standard procedure, it is obliged to test for malaria at every visit, because unlike HIV/AIDS that sufficient with one time test, for malaria, it’s not like everyday she got in touch with mosquitoes.” Head of puskesmas, MIMIKA  “I: What do you think about the malaria in pregnancy screening test perform on every ANC visit or IST? R: Do you mean we perform a screening test on every visit? I: Yes. R: I think it is a good idea, just in case the first time she visited there was no indication of malaria, but the next visit is already infected malaria then we can continue it to the medication. I think to keep performing the screening test is a good idea.” Village midwife, SUMBA |
|  | Women are happy to be screened for malaria* | “I: As a midwife, how do you decide that the pregnant woman needs malaria screening in the first visit of ANC? R: Usually, we give counselling about the benefit of malaria screening to pregnant woman. As I know, most pregnant women already know about what malaria is and what risk they will get. Most of them don’t mind to be screened for malaria.” Midwife coordinator, MIMIKA  “I: Do they ask about this blood test? R: Pregnant women who have good educational background often ask about the procedures, however for pregnant women who come from villages, they don’t ask, even they are happy to be blood taken, so they can know about the sickness.” Trial ANC supervisor, SUMBA |
| **Minor themes** | Some women may not like monthly testing* | “I: How do you think if the pregnant woman will be screened for Malaria every time she comes here for ANC visit? R: If we do screening to pregnant woman in every ANC visit, I think the pregnant woman will mind about that. As long as the pregnant woman has no malaria symptom, I think she doesn’t need to be screened.” Pharmacist, MIMIKA |
|  | Asymptomatic screening at first visit is sufficient | “I: Okay, so what do you think about performing a screening test on a pregnant woman at every ANC? R: Ouch, I don’t how thing goes in the back… the most important is… I: what do you think about it? R: the most important is at the first visit. For the next visit, it is only if there are medical complaints.” Malaria coordinator, MIMIKA |
| **RDTs** |  |  |
| **Major themes** | Results are not always accurate | “I: Was there any problem that you got to use RDT? R: I think the RDT result is not accurate. The microscope method is better than RDT. But the problem is we don’t have laboratory at this Puskesmas [health centre]. Maybe Eijkman is willing to provide the RDT to help us especially for malaria examination to pregnant woman.” Doctor, SUMBA  “I: what do you think about the RDT examination compare to the slide? R: Mm… as I said before, the program is going quite well. But when we screened the patient who has fever with RDT and the result was negative, we would crosschecked the result with slide.” Malaria coordinator, MIMIKA |
|  | RDTs don’t detect all malaria species | “I: The level of the case is pretty high, right? R: Yes, it is. I: But so far, the most appear, as the result of RDT test is malaria falciparum, is that correct? R: Yes that’s true. For vivax or ovale can’t be detected.” Lab tech, SUMBA |
|  | Supply is not stable, frequent stock-outs | “I: Is there any problem so far in screening test using RDT method? R: The unavailability of RDT kit itself is a problem. I didn’t know what causes it.” Village midwife, SUMBA  “I: How about RDT? R: We don’t have RDT but when we had RDT there was no pregnant woman came to Pustu [health post]. I: So, you don’t use RDT for general patient? R: We use RDT for pregnant woman only but we don’t have RDT anymore. I: When did you have RDT at this Pustu? R: Last year. I: Last year?” Village midwife, MIMIKA |
|  | Fast and easy to use | “I: Is there any problem in malaria screening with RDT for pregnant woman? R: I think RDT examination is not difficult to be done. The result is very quick, accurate and easy to read by anyone.” Midwife coordinator, MIMIKA  “I: What do you think about the malaria screening with RDT? R: I think RDT is very effective because that is very simple to use. And we can train our college easily to use that.” Doctor, SUMBA |
|  | Good for use at village posts, where lab services/electricity not available | “I: Do you think that it is fine to use RDT for SST program at Posyandu [health post]? R: In my opinion, it is fine because it’s impossible to bring microscope to the field moreover not all posyandu has electricity. That’s why RDT is really useful for out building examination.” Malaria coordinator, MIMIKA  “I: what kind of malaria examination instrument for pregnant women that is good to be used in Pustu [health post]? R: based on the facility and worker, if we use automatic microscope we need an analyst. Because in here we do not have the facility and worker, even for KIA [ANC] we don’t have lights, I think RDT is the most practical.” Village midwife, MIMIKA  “I: What do you think about how the RDT examination is suppose to be done? R: I think it would be better if RDT examination could be done at Posyandu [health post] and Pustu [health post] because at Puskesmas [health centre] there was Laboratory for malaria examination with slide. Besides that there is no electricity at Pustu or Poskesdes [health post] so that malaria examination with slide is not possible to be done there.” Midwife coordinator, SUMBA |
| **Minor themes** | Prefer RDT over microscope | “I: far, is there any obstacle in using RDT? R: No obstacle. I: Concerning the test result, which one is the better, by RDT or by microscope? R: I think RDT is the best, if I’m not mistaken, it is about anti-gen, and it is a part of the malaria itself. So the RDT is better. If we use microscope, there could be human error, so I prefer RDT.” Doctor, SUMBA |
| **IPTp** |  |  |
| **Major themes** | Women should be tested before they are given antimalarials | “I: How do you think about giving anti malaria drug without screening? R: It is not allowed. The diagnosis of malaria has to be confirmed first before we give the drug. We can’t make diagnosis of malaria by bare eyes. We can’t adjust the diagnosis of malaria by the symptom because the symptom may refer to another disease.” Malaria coordinator, MIMIKA  “I: What do you say regarding giving out drugs without screening? R: I disagree. They should be screened, examined, then given the drugs because, although it has no impact to the fetus, but sometimes we also know, because it’s malaria drugs. Better yet, do the examinations first, if positive for malaria then take the drugs.” Midwife coordinator, SUMBA |
|  | Taking medication during pregnancy when there is no disease could cause harm | “I: What is your opinion about medicine delivery without malaria screening test before? R: We can’t give the medicine without screening. When the pregnant women are given medicine without screening, only by seeing the symptom, it will destabilize the infant. The prevention is important, however the medicine of malaria is dangerous if it’s given without prescription, particularly for pregnant women. We have to make sure whether the pregnant women have malaria or not by doing screening test.” Midwife coordinator, SUMBA  “I: You mean the pregnant woman is not checked for malaria but she is given the drug? R: I don’t agree with that. I: can you explain the reason? R: Ya. The patient is a pregnant woman we don’t know about the side effect of the drug that could affect the unborn baby moreover there is a lot of changes happened in the pregnant woman body like hormone changing that makes the pregnant woman feel uncomfortable.” Doctor, MIMIKA |
|  | Could increase drug resistance | “I: What do you think about the malaria drug prescribing without screening? R: My opinion of the prescribing without screening, if you ask me, is I disagree. I only agree if the prescribing is based on diagnosis; if it’s positive, prescribe the malaria drug; based on our experience, if the patient is feverish doesn’t mean they are malaria, because the screening will show negative; I’m afraid of drug resistance. So I approve of they’re given malaria drugs after first being screened.” Malaria coordinator, SUMBA  “I: What do you think about not performing any screening test in pregnant woman but giving them a drug instead? R: ah that’s not, I’m sorry, what drug? I: Without screening test, and just to be given a malarial drug. R: never, never... we never prescribe malarial drug without examination first, it may risk a drug resistance.” Doctor, MIMIKA |
| **Minor themes** | Prevention is a good idea, if the drug is safe | “I: What is your opinion about giving malaria medicines without screening first? Just like what they did yesterday in Posyandu [health post], they just gave malaria medicines to the pregnant women for prevention, without screening. R: Yesterday? I: Yes. R: They gave the malaria medicines to the pregnant women? I: Yes. R: Yeah, I think that’s good, to prevent malaria on pregnant women.” Midwife coordinator, SUMBA  “I: So do you agree with that or? R: What should I say? I: It’s fine. You are free to say anything you want. R: All right… as long as the drug is safe for the mother, I think it’s good for the mother. But if the drug is not safe for mother, I think we have to stop it. I: So you? R: If the drug is safe for mother and doesn’t give bad effect for her pregnancy, it will be fine.” Midwife coordinator, MIMIKA |
|  | Some women may not want to take drugs if they are not sick* | “I: What is your opinion about using malaria medicines for the purpose of prevention? R: I think that’s good for prevention. It depends on the women, because usually they do not want to consume the medicines if they have no malaria symptoms.” Midwife coordinator, SUMBA  “I: What do you think if there is malaria medicine delivery without screening? R: I think it is difficult to do that in this area. Because the citizen will not consume the medicine if they are not sick. Moreover, pregnant women come here to do ANC visit, so it is difficult to give medicine every week.” Village midwife, SUMBA |
|  | Some women in the IPTp arm refused 2^nd^ dose of IPTp | “I: For the second visit, the patients who are positively infected by malaria would get the drugs. For the IPT, they would get more drugs without examination unless there’s a complaint. Has it ever happen that the pregnant women refuse to take the drug? For the further visit? R: O... it has happened couple of times. But mostly those who refuse the drugs are the IPT, because they take it every month. On the other hand, the SST or IST for instance is infected by malaria; they would just take the drug. They are rarely refusing to it. It’s mostly the IPT…” Trial research assistant, MIMIKA |
| **Antimalarials** |  |  |
| **Major themes** | Effective treatment for malaria | “I: Any problem in DHP prescription? R: so far, there is none, DHP is tolerated by most people and has minimum side effect. So far, it is seldom to have nausea complaints from the patients. Patients feel more comfortable and quicker to recover after taking DHP.” Doctor, MIMIKA  “I: How about Darplex? Any complain? R: I think there is no complain about Darplex. Ee… For Darplex, we ask the patient to take it for 3 days only. Darplex is a new drug for Malaria. It is not like Kina [Quinine] or Chloroquine that should be taken longer than Darplex. Mostly, after taking Darplex for 3 days, the patient looks well and fresh. That’s why they like Darplex. Actually, Darplex also has side effect but because the patient has to take it for 3 days, the side effect becomes less.” Midwife coordinator, MIMIKA |
|  | Well tolerated by women | “I: do you have an issue or obstacles in the usage of DHA piperaquine or darplex all these time? R: What sort of issue? I: Problems…maybe there has ever a complaint from the moms who has ever drank the darplex, the malaria medication that they drank? R: Not until now there has never been a complaint…” Village midwife, SUMBA  “I: Mam, have ever pregnant women complaint about side effects of medicines like OAM and Kina [Quinine]? R: No, we never get complaint, not yet. I: There has been no complain from .... R: No. I: No one said about headache? R: No. No one. I: Never? R: No. Never.” Pharmacist, MIMIKA  “I: Ooo… how about the DHP? R: DHP is better than Kina [Quinine] especially for the side effect. I: But it will be given not in the first trimester of pregnancy, right? R: Right. After first trimester of pregnancy.” Doctor, MIMIKA |
|  | Some reported side effects of nausea, vomiting, dizziness | “I: About the side effects which probably happened? R: It’s different among pregnant women. There are some who get nausea, while others feel so sleepy.” Trial ANC supervisor, SUMBA  “I: What is the side effect of darplex? R: What we now is nausea, more nausea. I: What other than nausea? R: Sometimes dizzy, but more to nausea.” Village midwife, MIMIKA |
| **Minor themes** | Some refused to take subsequent IPTp doses | “I: For the IPTp, in the next visit when the drugs are given to the patients, maybe there are some patients having complaints about the drugs given? Or, is there any patients refuse to take the drugs? R: There are also some patients refused because in the first enrolment, they took the drugs. Then in the next month visit, they refused to take the drugs. We usually ask them why? They say “Ouch ... I was rather dizzy when I took the drugs for the first time at home. I was also vomiting all over again until it could not get my activities done.” Trial research assistant, MIMIKA |
|  | DP is hard and bitter | “I: What do they say about the drugs which are given? R: For IPTp arm, most of participants who say that the drug is bitter and “hard”. They sometimes ask whether the drug influences to their pregnancy or not? Is it dangerous to consume malaria drug during pregnancy? After seeing the drug, they say “it’s so big”. But, we explain again that the drug is not bitter, though it’s big enough.” Trial ANC supervisor, SUMBA |
| **Service delivery** |  |  |
| **Major themes** | Screening should be carried out at health posts, more accessible to women | “I: Do you think that Malaria screening with RDT is very effective to be implemented in every Posyandu [health post]? Or do you think that will be better if the patient should be referred to Puskesmas [health centre] for malaria screening? R: I think that will better if the Malaria screening could be done in every Posyandu. It's cheaper than the pregnant woman has to go to Puskesmas for that because they don't need pay for transportation.” Village midwife, SUMBA  “I: do you think which one is better to perform a screening test at? Is it in Posyandu [health post] or in Puskesmas [health centre]? R: I prefer in Posyandu, mbak, because it is more concentrated. The problem in Puskesmas for pregnant women is the distance between their residence and Puskesmas. Those lives far from Puskesmas are very seldom to come to Puskesmas, they go to Posyandu instead. I prefer in Posyandu, because we got to interact with people.” Village midwife, MIMIKA |
|  | Midwives should use RDTs at health posts to screen for MiP | “I: In the guidelines, who is in charge to diagnose the patients with RDT kit? The lab workers or the midwives? R: Actually, in the guidelines, anyone can do that, as long as they know how to examine the patients by using RDT kit and they have trained in the provincial level. We told that to the fellow workers in Puskesmas [health centre] that the nurses, midwives can do that. Because if we keep insisting on placing the lab workers in Puskesmas, then will be in trouble because the lab workers are still hard to find, moreover in Pustu [health post].” Midwife coordinator, MIMIKA  “I: Do you have your own opinion or different intervention from Eijkman? R: Oh, I think it’s great. It’s great because they get close with the society, I mean midwife in this puskesmas [health centre] only know the area around here. They will go here if they are really sick, but their first contact is with the midwife in the village where the pregnant women or people with malaria live, so the approach by midwife in village is better and faster. That would be also good if they want to do malaria screening test directly in villages, so puskesmas only do coordination with them.” Malaria coordinator, SUMBA |
|  | Midwives should be able to give antimalarials to women when necessary | “I: do you have opinions regarding the policy that states a midwife may give medical prescription to the patients infected with malaria as discovered by malaria screening test? R: I think it is okay for the midwife to deliver therapy, because she knows about it, that a pregnant woman may consume malarial drug at certain month of gestational age. I think she understands that, it should not be a problem for a midwife to deliver treatment.” Head of puskesmas, MIMIKA  “I: In your opinion, which would be better if RDT test is conducted in posyandu [health post] or the patient is referred to puskesmas [health centre]? R: I think it is better if it is on posyandu. The important thing is the medicine is provided because if we do the test in posyandu and ask to take the medicine in puskesmas, I think they will not go to puskesmas to take the medicine. So it will be good if the test is conducted in posyandu and there is a stock of medicine.” Village midwife, SUMBA |
| **Minor themes** | Drugs should only be prescribed by doctors or nurses under supervision | “I: What do you think about the pregnant woman who comes for ANC visit should get blood examination at KIA and if the result is positive she will be given the drug by midwife? R: In my opinion, that is not quite efficient because all prescriptions that have been made should be sent to Pharmacy because we have to make a report for that so that we could know how many drugs we have used. So I think it is not efficient if the midwife give the drug.” Pharmacist, MIMIKA  “I: I mean, can a midwife give malaria medication for pregnant women? R: Em... maybe…. only me get this question? I: Yes. R: E’e.. So far, if there is a doctor, it is better the doctor give the medication. Because we also consider other factor, not only the medication. We can see if it is easy malaria case or difficult malaria case with complication. Complication sometimes needs further treatment and not only medicine. I: So, is it better that doctor is the one who give the medicine? R: yes.” Doctor, MIMIKA |
