# Supplementary material for: Intermittent screening and treatment or intermittent preventive treatment compared to current policy of single screening and treatment for the prevention of malaria in pregnancy in Eastern Indonesia: acceptability among health providers and pregnant women
Source: Malar J. 2018 Sep 27;17:341. doi: 10.1186/s12936-018-2490-3 (PMC6161378; doi:10.1186/s12936-018-2490-3)
Supplement: Supplementary file 2 — Additional file 2. Quotes from FGDs pregnant women on major and minor themes”. Quotes from pregnant women to support the analysis of the themes and sub-themes presented in the results. [file 12936_2018_2490_MOESM2_ESM.doc]

Additional File 2: Quotes from pregnant women on major and minor themes

| **Theme** | **Pregnant Women** | **Quotes** |
| --- | --- | --- |
| **SSTp** |  |  |
| **Major themes** | Happy to be screened for malaria | “I: Mrs. M? What did you like best in your first coming to this research? P2: I was happy because I can have malaria test and also fully blood check. I: Was there anything you don’t like? All: Nothing.” SSTp positive, MIMIKA  “I: So, from here (vein) and then finger tips. Okay I would like know what did you feel, Mama X, when your blood was drawn, how did you feel? P2: I’m glad because blood was drawn for medical purpose. Malaria test.” SSTp negative, SUMBA |
| **Minor themes** | Would prefer ISTp over SSTp | “I: Did you expect another blood obtaining or was the first visit enough? P4: Well we wanted to keep our blood obtained P1: I wanted to have my blood obtained. If that’s allowed by the officer we wanted to be examined all the time. We wanted to know our health, was there any malaria or not. In the upcoming months who would’ve known if we had malaria. I: I want to ask, on the first visit, you didn’t show any symptom of malaria, you weren’t sick, but your blood was tested. On the next visit, months afterward, right, Mama Y, they would draw your blood only if you are sick, do you agree? P2: I agree. To see if I’m healthy or not.” SSTp negative, SUMBA |
| **ISTp** |  |  |
| **Major themes** | Happy to be tested & know malaria status | “I: What did you feel when your blood was drawn? P8: I was shocked. I: Do you like blood drawing from your finger or not? P8: Yes, I do because we know that we got no malaria. Should we have malaria, then the drugs will be given. Should the malaria not be found in our system then we are just happy because we check for it monthly.” ISTp positive, MIMIKA  “I: What did you feel when you were told the result? P5: I was quite happy because there was no malaria in my system. P3: I was quite happy for having no malaria, because malaria is a common thing in Papua, moreover in Timika. People said that if you have never had malaria, then you have never been in Timika.” ISTp negative, MIMIKA  “I: So, you weren’t feeling afraid to have the blood test every month? P5: Yes. I was all right. The most important thing is healthy. I: How about you Mrs. D? P3: Same, I didn’t feel afraid.” ISTp positive, MIMIKA |
|  | Happy to be tested monthly | “I: Well I want to ask, for example, blood obtaining only at the first visit to posyandu [health post], after that there will be no malarial checkups, they will draw blood on the next month only if there is a complain. Now I’m comparing it with the previous one, blood obtain each month, every visit despite complaint. Which one do you prefer? P2: I prefer to obtain blood each month without preceded by complaint. I: Why is that? P2: Because we wouldn’t know if catch a disease this month or not.” SSTp negative, SUMBA  “I: So, in case you get pregnant later in the future, would you still agree that your blood is tested in every month? All: Agree I: Why do you agree mama A? Mama A has the most spirit in stated her agreement. P4: So I know whether I am infected by malaria or not. And to know the health of my baby as well.” IST negative, SUMBA  “1: How do you feel if each month your blood is taken for the check up? All: We’re happy. P3: Because we know that each month, oh it means we don’t have malaria.” SSTp negative, MIMIKA  “P1: I was so happy because I could know how my HB [hemoglobin] is every month and every month too we know whether we had malaria or not.” ISTp positive, MIMIKA |
|  | Happy to be tested even when asymptomatic | “I: Well when you first came, you weren’t sick, you were pregnant, but not sick. But you were tested for malaria; do you want it that way? All: I do! P2: I do because they said it was to see if I have malaria or not. I: So, you prefer to have your blood drawn even though you weren’t sick, or to get sick first and then have your blood drawn? P1: Before I get sick, of course. P2: Before I get sick P5: Before I get sick.” SSTp negative, SUMBA  “I: What is your opinion, if in the beginning you have all checked, but in the next coming you’ll be only checked if you have the signs? What do you think? P1: For me, it is better to have checked even there is no sign because I want to know. If they ask me to do, I’d love too even there is no indication.” SSTp positive, MIMIKA |
| **Minor themes** | Prefer testing only when symptomatic | “I: And Mama Y, you aren’t sick, but your blood is drawn in order to check on malaria, do you agree to that? P2: I disagree. I have to be sick first to have blood test to find out what kind of sickness I’m having.” ISTp negative, SUMBA  “I: What about you Mrs. F? P7: I’d prefer to have the test if I have the signs or indications like fever because I have once had blood taken but no malaria….I: What about you Mama D? P3: I usually have test if I have fever, if there is no sign, then I will not do the test.” ISTp positive, MIMIKA |
| **RDTs** |  |  |
| **Major themes** | Happy to receive results right away | “I: And what about mama K, which tool? P5: The rectangular one. I: The rectangular the RDT one? P5: Yes I Was it showed to you? P5: Yes it was showed and they said my result was negative, not positive. I: Were you happy or not happy when your malaria test result is out? P1: I was happy. I: Why was that? P5: Because there was no malaria on the baby I: Anything that you dislike? P7: Nothing, I guess.” ISTp negative, SUMBA  “I: The result is known from the test pack you mentioned, uhm…RDT. But there is also the result presented from the glass, I ask you, which one do you prefer to get the result from? P3: The direct one. Yes, RDT. I: Anything else? P2: I think the result came up faster by using that, so our curiosity will be swiftly vanished, we don’t need to spend long time just to wait for the result.” ISTp negative, MIMIKA |
|  | Don’t mind the finger prick | “I1: But before they took your blood, pinned with the needle; how did you feel when they did it? Were you afraid? P5: Afraid, I was afraid. They took my blood in here (finger’s edge) and in here (vein’s blood). I felt no fear when they took it here (finger’s edge), I felt fear if they took it here (vein’s blood).” Heterogeneous, MIMIKA |
| **Minor themes** | Afraid of needle/blood loss | “I: Mama, when you had blood test, in the first time and also the next, did you feel afraid? P1: I do afraid, shocked. I was afraid the needle.” ISTp positive, MIMIKA  “I: The mothers come without feeling pain, but the bloods are taken. How about that? P1: So I’m afraid of the needle, so I will ask why I have to be checked although I’m healthy. There is the feeling like it’s not necessary, it’s not needed. But I’m happy when I get a negative result.” SSTp negative, MIMIKA |
| **IPTp** |  |  |
| **Major themes** | Happy to take drugs to prevent malaria & be healthy (even with side effects) | “I: So, you agree if you were given meds without being sick. Without examination, without you having malaria, you were given meds to take. P3: Yes, I agree, in fact it is better. Prior to giving the meds, the doctor told me that this is to eliminate malaria, so the doctor had explained to me before giving them, so when it was given to us, we accepted it wholeheartedly, although there was some hesitation. P2: Even though there was no sickness, they said this is malarial drug, for prevention, we’re glad to take it.” IPTp, SUMBA  “I: So, you took the medicine as you knew that it’s for malaria defensing? P2: Yes, because we knew it’s for malaria defensing, more for our baby. Moreover, if my mom also there yeah I had to take the drugs. I: So for the next pregnancy, will take the drugs to defense malaria? All: Yes, I will. I: What makes you will? P5: Yeah, we want it, to keep me and my baby’s healthy and free from malaria. I: What about if you still have dizzy? P5: That’s alright. We have to fight for mother and baby’s healthy.” IPTp, MIMIKA |
|  | Will take the drugs when reassured there is no harm to baby, given by trusted provider | “I: So how did you feel when you got to take the medicine in the second visit? P5: The first is for my health so I got to take it, they would not give me the medicine I don’t need so, if they give me the medicine, I would take it.” IPTp, SUMBA  “I: What was your opinion at the time? You haven’t known yet whether you had malaria or not? But they gave you drugs, how did you feel? P3: One thing that I thought at the time was they want to give the drugs, if that was not for my goodness they wouldn’t give them to us. So, I thought that it was for my goodness, my healthy so I took the drugs.” Heterogeneous, MIMIKA |
| **Minor themes** | Prefer testing before taking drugs | “I: Before you were had malaria test first and then had the drugs, hadn’t it? What do you think about that? P6: I am not so happy with that, I’d prefer having check and has no malaria but they still give us malaria defensing drugs.” Heterogeneous, MIMIKA |
|  | Hesitant at first to take drugs but did so for baby’s health | “P2: For me, I had to think it again and again, I would queasy and I was thinking to throw the drugs away. Every time I took the drugs, my mom always there and encouraged me by saying ‘think about your baby’ then I took the drugs as they are good for me and my baby.” IPTp, MIMIKA |
| **Antimalarials** |  |  |
| **Major themes** | Experienced side effects of nausea, dizziness, sleepiness | “I: Mama D and Mama Y, just speak in local language if you don’t speak Bahasa Indonesia very well. Okay, anything you dislike? P3: Nothing, I like it in fact. P2: Nothing. It’s just that when I took the medicine, the three pills at once, I felt sickness immediately. But that’s what the effect they said.” IPTp, SUMBA  “I: Well. How about you Mrs. M? P7: They gave the drugs, before that they checked my blood and result showed I had no malaria. I asked the nurse, how it is. Then she said no problem, the drugs have a low doses and then I take 3 tablets. I got dizzy in the first and third days and then I went to asleep. In the second day, I had the drugs I felt nothing.” IPTp, MIMIKA  “I: How many time you had malaria drugs while joining the program? P3: I got them every month. After 5th month up, so I got them since my pregnancy was 6th till the 9th. So, I got for 3 months continuously. I: What do you think about having drugs every month? Don’t you aware the effect, do you? P3: No problem, I am happy. Obviously, I felt dizzy and also queasy; I know that’s the effects. If we don’t have enough rest then we would be queasy and dizzy. But Alhamdulillah I am healthy.” Heterogeneous, MIMIKA |
|  | Completed my treatment/IPTp dose | “I: Did they give you malaria drugs? P9: Yes, they did. I ate the medicine in the Posyandu [health post] at the time. I: How many did you eat? P9: Three tablets and I brought the rest home. I: How many days did you spend the drugs? P9: The first day was in Posyandu, two days forward at home. Then I brought back the place. I: When you took the drugs home, how did you eat it? Did you eat it yourself? P9: I ate it myself.” IPTp, MIMIKA  “I: When it’s time to take the medicine at home, did you do it yourself or did you have anyone reminding you to take the medicine? P1: I took it myself, because sometimes when I forgot, there would be somebody to remind me. My husband is the one who reminds me to take the medicine. If I had fallen asleep, he would wake me up to take the medicine.” IPTp, SUMBA |
| **Minor themes** | Experienced vomiting | “I: Mrs. R, how was your experience? P3: I didn’t have the drugs at all doctor. I threw up them all. I guess it because I was in ngidam period, I threw them all. Then I called the nurse, I explained her that I threw all the drugs up, after 5 minutes. I watched the time so I knew I threw all the drugs up after 5 minutes. They drugs were invisible on the vomit, so I assumed that the drugs were already digested. Then the nurse advised me to reduce the doses, but I still threw them up.” IPTp, MIMIKA  “I: Mrs. M, how was your second and third days? Did you feel queasy? P6: Yeah, I still felt that. The drugs are too bitter for me. P7: For me, on the second day I ate the drugs after eating. I ate till full then had the drugs. On the third day as I ate the drugs I felt nothing. I: Mrs. MA, what about you? P1: As same as Mrs. N, on the second and third days I ate before having the drugs. I felt nothing, no queasy, and then I went to asleep. P2: On the first day, we didn’t eat, we went to Puskesmas [health centre] directly ate the drugs without eating first so we felt queasy and dizzy.” IPTp, MIMIKA |
|  | Did not complete my treatment/IPTp dose | “I: How about you Mama S? P9: The long one, if I took the long one I felt queasy. My husband asked me to drink more water but then I threw them all out, then I drink it more. I: Why? Was it caused by the size or how? P9: That was too big for me, the circle one.” IPTp, MIMIKA  “I: What about you Mama M? P7: They gave me 3 times, the third one I didn’t take them. My husband got Malaria and he took the drugs randomly. He ate paracetamol in the night, he took it when he was in work place, then as he went down he said he felt fever still. He asked me for malaria drugs, I asked to go to Puskesmas [health centre] he didn’t want. Then I gave him that malaria drugs, I became doctor. Hahahahahahaha… the drugs must be for me I gave him, I helped my husband. Hahahahahaha...” IPTp, MIMIKA |
